# Gastrointestinal Stromal Tumors (GISTs) in Pediatric Patients: A Case Report and Literature Review

**DOI:** 10.3390/children11091040

**Published:** 2024-08-26

**Authors:** Tudor-Alexandru Popoiu, Cãtãlin-Alexandru Pîrvu, Cãlin-Marius Popoiu, Emil Radu Iacob, Tamas Talpai, Amalia Voinea, Rãzvan-Sorin Albu, Sorina Tãban, Larisa-Mihaela Bãlãnoiu, Stelian Pantea

**Affiliations:** 1 Department of General Surgery, “Victor Babeş” University of Medicine and Pharmacy, 300041 Timisoara, Romania; popoiu.tudor@umft.ro (T.-A.P.); ama_voinea@yahoo.com (A.V.); albu.razvan96@yahoo.com (R.-S.A.); pantea.stelian@umft.ro (S.P.); 2 Department III of Functional Sciences, Discipline of Medical Informatics and Biostatistics, “Victor Babeş” University of Medicine and Pharmacy, 300041 Timisoara, Romania; 3 Department of Pediatric Surgery, “Victor Babes” University of Medicine and Pharmacy, 300041 Timisoara, Romania; mcpopoiu@umft.ro (C.-M.P.); radueiacob@umft.ro (E.R.I.); 4 Department of Pathology, “Victor Babeş” University of Medicine and Pharmacy, 300041 Timisoara, Romania

**Keywords:** pediatric GIST, quadruple negative, tyrosine kinase inhibitor, massive hemorrhage, emergency surgery

## Abstract

Gastrointestinal stromal tumors (GISTs) are rare mesenchymal neoplasms that primarily affect adults, with pediatric cases constituting only 0.5–2.7% of the total. Pediatric GISTs present unique clinical, genetic, and pathological features that distinguish them from adult cases. This literature review aims to elucidate these differences, emphasizing diagnostic and therapeutic challenges. We discuss the resistance of pediatric GISTs to conventional chemotherapy and highlight the importance of surgical intervention, especially in emergency situations involving intra-abdominal bleeding. The review also explores the molecular characteristics of pediatric GISTs, including rare mutations such as quadruple-negative wild-type GIST with an FGF3 gene gain mutation. To illustrate these points, we conclude with a case from our clinic involving a 15-year-old female with multiple CD117-positive gastric GISTs and a quadruple-negative wild-type genetic profile who required urgent surgical intervention following a failed tumor embolization. This case underscores the critical need for early diagnosis and individualized therapeutic strategies combining oncologic and surgical care to improve outcomes in pediatric GIST patients.

## 1. Introduction

Gastrointestinal stromal tumors (GISTs) are the most prevalent mesenchymal neoplasms of the gastrointestinal tract, with an annual incidence of 5000 to 6000 new cases in the United States [1], with a reported increase in incidence [2]. These tumors originate from the interstitial cells of Cajal, which are integral to gastrointestinal motility [3]. Most gastrointestinal stromal tumors (GISTs) are found in older adults, and they are typically driven by activating mutations in KIT or PDGFRA receptor signaling. However, 10–15% of GISTs do not have these mutations and are referred to as wild-type. These wild-type GISTs (WT GIST) fall into three clinic-pathologic subgroups: pediatric GISTs, those associated with neurofibromatosis type 1, and sporadic wild-type GISTs [4]. Pediatric GISTs, though rare, present a significant clinical challenge due to their differing behavior compared to adult GISTs and their resistance to standard tyrosine kinase inhibitor (TKI) therapies.

Pediatric GISTs often manifest in the stomach and are predominantly wild-type for KIT and platelet-derived growth factor alpha gene (PDGFRA) mutations, distinguishing them from their adult counterparts, which frequently harbor these mutations. These tumors are also noted for their tendency to metastasize to lymph nodes, liver, and peritoneum, as well as their association with succinate dehydrogenase (SDH) deficiency, leading to distinct molecular and epigenetic profiles [5,6].

The rarity of pediatric GISTs complicates the establishment of standardized treatment protocols, necessitating a reliance on case studies, retrospective analyses, and small clinical trials. Traditional management strategies, primarily developed for adult GISTs, have limited efficacy in pediatric cases, highlighting the urgent need for tailored therapeutic approaches.

This review aims to consolidate current knowledge on pediatric GISTs, encompassing their epidemiology, molecular biology, clinical presentation, diagnostic modalities, and treatment options. By synthesizing the latest research and clinical findings, this review seeks to provide a comprehensive overview that will inform clinical practitioners and guide future research efforts in the management of pediatric GISTs. Moreover, a subsequent case report challenges the data presented, emphasizing the importance of considering what usually seems to be an indolent tumor as an acute manifestation that requires effective collaboration across various specialties.

## 2. Epidemiology

The largest series presented to date revealed that the percentage of patients with GIST below the age of 21 years ranged from 0.5% to 2.7% [7]. The age of onset for pediatric/WT GIST is notably younger, with a median diagnosis age in the second decade of life, compared to the median age of 63 years for adult GISTs. Additionally, there is a notable female preponderance in pediatric GIST cases, with approximately 70% of diagnoses occurring in females, whereas adult GISTs show an equal gender distribution [8]. 

Before the introduction of tyrosine kinase inhibitor (TRKI) chemotherapy, adult studies reported approximately 50% 5-year survival rates for GIST. Since 2000, these rates have improved to up to 80%. Notably, pediatric GIST patients now exhibit over 90% 5-year survival rates, surpassing many adult cases. While short-term follow-up in some instances could introduce bias, Ritio’s study’s median follow-up period of 6.7 years is significantly longer than that of most pediatric studies [9].

## 3. Diagnostics

### 3.1. Clinical Features

The submucosal localization of GISTs carries the tendency of a long asymptomatic evolution in adult patients. For pediatric patients, the usual clinical presentation is gastrointestinal bleeding (melena or hematemesis), and its secondary chronic anemia, paleness, vertigo, and fatigue, mainly due to its predisposition of gastric localization (90% of pediatric GISTs [9] vs. 60% of adult GISTs [10]). Other frequently encountered symptoms may be abdominal pain that might appear suddenly, such as an acute abdomen or chronically manifesting nausea and vomiting, intestinal obstruction, or constipation and palpable abdominal masses [11,12]. Pediatric GISTs are primarily found in the stomach, unlike the wider range of locations seen in adult GISTs. They are infrequently located in the small bowel, appendix, Meckel’s diverticulum, mesocolon, rectum, or retroperitoneum [13,14,15]. Notably, pediatric/WT GISTs tend to involve lymph nodes, liver, or peritoneum metastases at diagnosis, occurring in approximately 45% of cases [8,16]. The natural history of this disease manifestation is different for the adult and pediatric types as well: a 50% 5-year survival rate and a 50% recurrence or metastasis presence in adults vs. a more slow-going disease course in pediatric patients despite the high metastatic rate at around 65% [17,18,19].

### 3.2. Pathology

Morphological and immunohistochemistry studies are used in the pathological diagnosis of gastrointestinal stromal tumors (GISTs). Spindle cells (70%) and epithelioid cells (20%) are the most common cell types [20]. Ninety-five percent of GISTs have positive KIT (CD117) and/or DOG1 immunohistochemistry, and seventy percent additionally have positive CD34 immunohistochemistry. KIT positivity is an important diagnostic characteristic, but without complementary morphological traits, it is inadequate on its own. Staining with CD34 and DOG1 is utilized for KIT-negative patients. Furthermore, about 80% and 10% of GISTs, respectively, have mutations in KIT or PDGFRA. It is advised to use immunostaining for succinate dehydrogenase subunit B (SDHB) in patients lacking these mutations, especially in stomach GISTs [21]. Interestingly, pediatric GISTs show elevated mitotic activity. The median mitotic index was 6 in patients with favorable outcomes, and it was significantly higher, at 48, in those who did not survive. In pediatric GISTs, epithelioid morphology is the most common, with spindle and mixed tumor morphologies being equally distributed. Unlike findings in the adult GIST population, spindle cell morphology in pediatric patients is linked to a poorer prognosis [9].

### 3.3. Genetics and Genotyping

**Figure 1:**
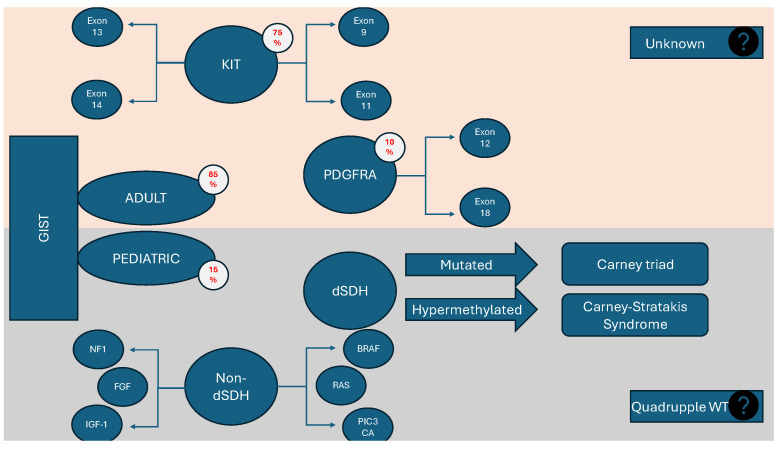
Molecular characteristics of adult and pediatric GISTs.

The crushing majority of adult GISTs (90%) encompass a set of modifications corresponding to certain mutations of two genes: KIT and PDGFRA. In the majority of GISTs, KIT, a receptor tyrosine kinase (RTK), is highly expressed and often mutated. When KIT is wild-type, most instances have activating mutations in PDGFRA, another RTK. For GIST cells to proliferate and survive, mutant KIT/PDGFRA must activate the Phosphoinositide 3-kinase (PI3K) pathway downstream. Furthermore, the activation of the Mitogen-activated protein kinase (MAPK) pathway signaling, which is also triggered via KIT, is essential for the growth of tumors since it stabilizes the transcription factor ETV1 and starts an oncogenic transcriptional program [22]. In addition to the preferential activation of the MAPK pathway, fibroblast growth factor receptor 3 (FGFR3) activation partially restores KIT activity. It has been presented as an interaction between KIT and FGFR3 in the context of their overexpression. Although data support a direct interaction between these receptors, it is just one potential mechanism underlying the crosstalk observed in GIST cells. Other possibilities, such as the involvement of downstream mediators, should also be considered [23]. GISTs without mutations in KIT or PDGFRA receptors are known as KIT/PDGFRA wild-type (WT) GISTs, comprising 10–15% of adult cases. Among these, 20–40% are SDH-deficient, lacking SDHB protein expression. About 15% have proto-oncogene B-Raf (BRAF)/Rat sarcoma (RAS) or Neurofibromatosis type 1 (NF1) mutations, termed RAS-pathway (RAS-P) mutant GISTs. The remaining KIT/PDGFRA WT GISTs, with intact SDH complexes, are referred to as quadruple WT GISTs. This group, making up 50% of KIT/PDGFRA WT and 5% of all GISTs, has a distinct transcriptome profile, indicating that they represent a unique GIST subgroup [24,25,26] (Figure 1). Pediatric GISTs necessitate detailed molecular classification using DNA and RNA sequencing and immunohistochemistry. Advanced methods like single-molecule molecular inversion probe (smMIP) technology and whole-exome sequencing (WES) enable the identification of specific mutations, guiding targeted therapy [27]. Approximately 85% of pediatric GISTs are wild-type (WT), often characterized by mutations or silencing of SDH complex genes, and they frequently exhibit neurotrophic tyrosine receptor kinase (NTRK) mutations or fusions [6,28]. Some GISTs also have fibroblast growth factor receptor (FGFR) abnormalities, which can be targeted with specific inhibitors to overcome resistance to treatments like imatinib, and combining FGFR inhibitors with standard chemotherapy may enhance treatment efficacy. In quadruple-negative WT GISTs, the overexpression of Fibroblast growth factor 4 (FGF4) is caused by the unique genomic structure of the duplication, rather than an epigenetic mechanism. Although different molecular processes lead to FGF4 overexpression, these results highlight the increasing biological importance of the FGFR pathway in WT GISTs, encompassing both SDH-deficient and quadruple WT forms. This indicates that the FGFR pathway might serve as a universal therapeutic target for these types of GISTs [26]. Additionally, mutations in RAS or NF1 genes are critical for predicting malignancy and targeting treatments. NF1-mutant GISTs, often found in the small intestine, have a good prognosis, necessitating the monitoring of children with RASopathies for early detection [6,29]. 

#### 3.3.1. Succinate Dehydrogenase-Deficient GISTs (dSDH)

A completely separate category of pediatric GISTs is represented by those with SDH deficiency, which, due to their phenotypic and genotypic particularities, pose the greatest challenge in terms of diagnosis and therapeutic management. A deficiency in the succinate dehydrogenase complex (SDH), whether due to loss-of-function mutations or the reduced expression of SDH-encoding genes, is associated with the majority of wild-type GISTs [30]. GISTs from patients with hereditary paraganglioma syndromes, Carney–Stratakis syndrome, Carney triad, and pediatric GISTs in general have been found to lack a certain component of the SDH (coenzyme Q reductase or mitochondrial complex II), the hydrophilic B subunit of SDH, leading to an unstable enzyme complex and the accumulation of succinate during the Krebs Cycle [31]. This represents a crucial step in the Krebs cycle or (tricarboxylic acid cycle) TCA cycle that governs carbon metabolism and energy production within cells. A deficiency in SDH alters cellular metabolism, affecting the requirement for extracellular pyruvate and the biosynthesis of aspartate and resulting in elevated succinyl-CoA levels [32]. Succinyl-CoA serves as a substrate for lysine succinylation, a common posttranslational modification influencing cellular stress responses. In tumor cells harboring isocitrate dehydrogenase (IDH) or SDH mutations, mitochondrial proteins undergo hypersuccinylation, leading to mitochondrial depolarization and altered metabolic profiles conducive to cancer progression. This hypersuccinylation also contributes to apoptosis resistance by facilitating the localization of the prosurvival factor B-cell lymphoma-2 (BCL-2) to the mitochondrial membrane [33,34]. Metabolites influence epigenetic modifying enzymes in two main ways. Firstly, enzymes like p300 can be activated through direct acetylation, affecting their structure and function. Secondly, metabolites such as α-ketoglutarate and oncometabolites (e.g., succinate, d-2-hydroxyglutarate (d-2HG), and fumarate) serve as cofactors or inhibitors of enzymes like α-ketoglutarate-dependent dioxygenases. Mutations in enzymes like SDH, IDH, and fumarate hydratase (FH) cause the accumulation of these metabolites, which competitively inhibit dioxygenases [35,36]. Succinate is considered an oncometabolite, being associated with inhibiting the α-ketoglutarate-dependent dioxygenase family of enzymes, which includes around 60 members that regulate crucial aspects of tumorigenesis and impacting various epigenetic processes such as DNA and histone demethylation, hypoxia responses, and mRNA modification [36]. Hypoxic disbalance is a well-described phenomenon of tumorigenesis. The activation of pseudohypoxia signaling via the overexpression of hypoxia-inducible factor 1 alpha (HIF1α) is considered a central event in the pathogenesis of SDH-deficient tumors [37]. Forkhead box protein M1 (FoxM1), a transcription factor that balances cell proliferation and differentiation, is highly expressed in aggressive GISTs, induced through hypoxia via both HIF-1α and HIF-2α, promoting cell proliferation, metastasis, and invasion [38]. Additionally, it has been demonstrated that silencing SDHB expression induces tumor-like phenotypic traits in cell cultures [4].

#### 3.3.2. BRAF and RAS Mutations

BRAF and RAS gene-family mutations represent a distinct subgroup of mutations that influence the development and treatment response of certain GIST subtypes. They were initially identified in triple-negative wild-type GISTs, and some studies have also found these mutations in conjunction with KIT/PDGFRA-mutated adult GISTs, which explains the lack of response to conventional imatinib treatment in these cases [39,40]. BRAF mutations, particularly the V600E mutation, result in the constitutive activation of the BRAF kinase, leading to continuous signaling through the MAPK/ERK (Mitogen-Activated Protein Kinase/Extracellular Signal-Regulated Kinase) pathway, promoting cell proliferation and survival. Similarly, RAS mutations, especially in NRAS and KRAS, cause the persistent activation of the MAPK/ERK pathway by promoting GTP-bound active RAS, which then activates downstream effectors, including RAF, MEK, and ERK. This continuous signaling drives tumor growth and survival [41,42,43].

Interestingly, some studies suggest that quadruple-wild-type GISTs with BRAF mutations have a worse prognosis and survival rate than succinate dehydrogenase (SDH)-deficient GISTs and exhibit increased resistance to imatinib, sunitinib, and regorafenib, with some susceptibility to sorafenib [44,45]. BRAF mutations occur early in GIST development and are present in very small tumors, with diameters as small as 4 mm [41,46]. These mutations are more commonly detected in the small bowel, and their clinical behavior is diverse [29].

### 3.4. Imaging

#### 3.4.1. Computed Tomography 

Computer tomography is the gold standard for imaging adult patients with GIST due to its high reliability in tumor detection and staging, rapid availability, and speed, with the option to improve tumor detection and staging and assess evolution through a multiphase computer tomography technique [21,47]. Typically, GISTs present as large (over 5 cm), exophytic, hypervascular tumors with heterogeneous enhancement in contrast-enhanced computer tomography due to necrosis, cystic degeneration, hemorrhage, or, rarely, calcification. Cavitation, ulceration, and fistulation to the gastrointestinal lumen are also common, while smaller tumors (under 5 cm) usually appear as submucosal or endoluminal polypoid masses with uniform contrast enhancement [48,49]. Moreover, positron emission tomography (PET-CT) is highly sensitive (90–100%) in detecting metastasis, and it enables the early assessment of therapeutic response to TKIs like sunitinib [50,51]. Still, computer tomography necessitates high radiation exposure, and although advanced devices can lower radiation output by 33%, the repeated imaging necessary for monitoring renders magnetic resonance imaging (MRI) and contrast-enhanced ultrasound (CEUS) are better indicated for pediatric use [52].

#### 3.4.2. Magnetic Resonance Imaging

MRI effectively identifies the primary tumor’s location and its relation to major vessels, which are essential for preoperative planning. It does not seem to be less accurate than computer tomography, but the imaging protocol can be more complicated, entailing the necessity of performing a series of sequences (T1, T2, diffusion-weighted imaging-DWI, contrast-enhanced imaging, and T1 fat saturation) [53]. Tumors’ appearance in MRI varies by size: limited tumors (under 5 cm) show round shapes with intense, uniform arterial enhancement, while larger tumors (over 5 cm) appear irregular with mild, heterogeneous enhancement and often contain intratumoral cysts. In adults, the presence of cysts or low apparent diffusion coefficient (ADC) values suggests high-risk GISTs. High cellular density restricts water movement, measurable via DWI and ADC values, which can indicate malignant potential [54,55].

#### 3.4.3. Contrast-Enhanced and Endoscopic Contrast-Enhance Ultrasound

Contrast-enhanced ultrasound (CEUS) uses blood-soluble contrast agents to evaluate hypervascular and hypovascular tissues effectively. It is particularly useful for assessing GISTs, which are hypervascular, and monitoring changes in tumors’ vascularity and structure following TKI treatment. CEUS can detect treatment responses, as it is more sensitive than PET-CT in identifying small hypovascular lesions [56,57]. The option for endoscopic use improves sensitivity, and other techniques, such as elastography, can further be integrated into the echographic protocol [58]. Recent studies have indicated that contrast-enhanced EUS is highly effective in detecting perfusion patterns, showing greater sensitivity for identifying neovascularity within lesions compared to endoscopic ultrasound–fine-needle aspiration (EUS-FNA), contrast computer tomography, and Doppler EUS. Although the prognostic significance of neovascularity in GISTs needs further investigation, this technique presents a promising new method for diagnosis and monitoring treatment [59,60]. It is advantageous because of its low operating cost, repeatability, and lack of nephro- and hepatotoxicity, but it is very operator-dependent and thus harder to standardize.

### 3.5. Endoscopy

In pediatric patients, endoscopy is frequently employed when there is a suspicion of an obstructing mass or gastrointestinal bleeding, which are common signs of GISTs. GIST lesions usually manifest as smooth-bordered, submucosal masses that cause the mucosa to protrude above. Necrosis or bleeding are not commonly associated with GIST lesions. Concurrent endoscopic ultrasonography (EUS) can direct tissue sampling and assist in differentiating between intramural and extramural lesions [58,61].

### 3.6. Biopsy

About one cubic centimeter of tissue, or 5–10 core-needle biopsy specimens, is required for the diagnosis of GISTs. The biopsy has to be done deeply enough to obtain representative tissue since the tumor resides submucosally. The best way to collect these samples is via endoscopy, using forceps biopsy (FB) or core-needle biopsy (CNB), with or without ultrasound guidance. This preference stems from the possibility of tumor spilling, a danger associated with percutaneous image-guided or surgical biopsies and linked to recurrence in juvenile GISTs. Primary resection can be a wise substitute for endoscopic biopsy in cases when anatomical limitations prevent it from being possible to acquire a tissue diagnosis. Percutaneous image-guided methods may be preferable for sampling metastatic lesions even if they are often avoided for initial lesions [62,63,64].

## 4. Associations with Other Pathological Entities

### 4.1. Carney Triad and Carney Syndrome

Carney triad (CT) is a rare condition characterized by the presence of at least three types of tumors: gastric gastrointestinal stromal tumor (GIST), paraganglioma (PGL), and pulmonary chondroma. It predominantly affects females and is not inherited. The cause of CT (Carney triad) is not yet clear, but recent studies suggest an involvement of the Succinate dehydrogenase complex subunit C (SDHC) gene. Haller et al. found that specific DNA hypermethylation in the SDHC gene’s promoter and first exon reduces SDHC mRNA expression in CT patients. This hypermethylation is now recognized as a molecular signature of CT and is used diagnostically to identify suspected CT lesions [65,66,67,68]. Carney–Stratakis syndrome (CSS), or dyad, shares two of these tumor types (GIST and PGL) and affects both genders during childhood and adolescence. Unlike CT, CSS follows an autosomally dominant inheritance pattern with incomplete penetrance. Both syndromes are linked to a deficiency in the succinate dehydrogenase (SDH) enzyme, but they differ in their underlying molecular mechanisms. CT typically involves site-specific hypermethylation of the SDHC gene, leading to SDH downregulation, while CSS involves inactivating germline mutations in one of the SDH subunit genes (SDHA, SDHB, SDHC, or SDHD) [66]. Despite some common features between CSS and CT, the prevalence of shared tumors differs: paraganglioma is more common in CSS, while GISTs are more prevalent in CT. Notably, in most patients with GISTs, the sarcoma presents with symptoms before the development of paragangliomas or pheochromocytomas [66,69].

### 4.2. Neurofibromatosis Type I 

Neurofibromatosis type 1 (NF1), also known as von Recklinghausen’s disease, arises from mutations in the NF1 gene spanning over 350 kb on chromosome 17q11.2. The NF1 gene encodes neurofibromin, a member of the GTPase-activating protein (GAP) family involved in regulating Ras proteins. The mutations in NF1 are highly diverse, and diagnosis is primarily based on clinical criteria. Gastrointestinal manifestations of NF1, although less common than neurocutaneous symptoms, include various lesions, such as hyperplastic intestinal neural tissue, gastrointestinal stromal tumors (GISTs), duodenal and periampullary endocrine-cell tumors, and other miscellaneous tumors. GISTs are the most common non-neurological tumor observed in NF1 patients, often presenting with multiple tumors being prone to more resistance against imatinib treatment [70]. However, the histological and immunohistochemical distinctions between GISTs in NF1 and non-NF1 patients remain incompletely understood [71,72,73].

## 5. Risk Stratification

Risk stratification is mainly based on tumor size, mitotic count, the anatomical location of the primary tumor, and tumor rupture [74,75]. It is difficult to distinguish benign from malignant GISTs using only tumor diameter or the mitotic index. Nomograms and risk categorization were, therefore, introduced. Because of its historical background, the NIH classification—which is based on tumor size and the mitotic index—is often used in treatment studies [76]. The Miettinen and Lasota approach, which takes tumor size, mitotic count, and location into account, is often applied in clinical practice. When choosing patients for adjuvant treatment, the “modified NIH classification”, which takes into account mitotic count, size, location, and rupture, may offer additional guidance [21,76,77]. Recently, transcriptional determinants such as HAND-1 or BARX-1 overexpression were correlated with increased metastatic rate and overall tumor aggressivity [78]. Still, WT/pediatric GISTs embody such a different phenotype that it is hard to include them in the same risk categorization as adult GISTs, with a more careful analysis of every individual case being necessary.

## 6. Treatment

The most valid approach to managing pediatric GIST patients should comprise a team of specialists made up of pediatricians, oncologists, pediatric surgeons, surgical endoscopists, and radiologists evaluating every step from initial presentation to follow-up. The primary distinction in treating pediatric GISTs compared to adult cases lies in their increased resistance to chemo- and immunotherapy and the higher propensity for disseminated disease. This makes management more challenging and often requires more extensive surgical intervention, deviating from the more standardized treatment protocols used for adult GISTs. 

### 6.1. Medical Treatment

Pediatric GISTs present unique challenges in their medical management, primarily due to their distinct biological and molecular characteristics compared to adult GISTs, the absence of KIT or PDFGRA mutations making them less susceptible to the adult standard tyrosine kinase inhibitor-based therapy. The rarity of pediatric GISTs, along with their differing response to conventional therapies and the lack of response to conventional chemotherapy or radiotherapy necessitates a tailored approach to treatment [74].

The cornerstone of medical treatment for GISTs in adults has been the use of tyrosine kinase inhibitors (TKIs), particularly imatinib. Approximately 80% to 90% of patients with unresectable or disseminated GISTs initially achieve at least disease stabilization, or a complete or partial response, with imatinib mesylate. However, nearly 50% of GIST cases treated with imatinib develop secondary resistance within the first two years. This secondary resistance is most frequently due to the acquisition of additional mutations in KIT or PDGFRA, which reduce imatinib’s binding affinity. Additionally, another mechanism that likely contributes to acquired resistance in a subset of GISTs is the activation of alternative pathways, bypassing the inhibitory effects of KIT/PDGFRA-targeted therapies [79,80]. This breakthrough has established GISTs as a model for precision medicine, leading to the adoption of TKIs as the standard treatment for this chemo-resistant and rare tumor [81]. However, pediatric GISTs often exhibit mutations that make them less responsive to imatinib [82]. Most pediatric GISTs lack the common KIT and PDGFRA mutations seen in adult cases, with a significant number being wild-type for these genes. Instead, many pediatric GISTs are characterized by succinate dehydrogenase (SDH) deficiencies, due either to mutations or epigenetic modifications, which necessitates alternative therapeutic strategies [83].

Imatinib remains the first-line therapy for both KIT/PDGFR-mutant and wild-type pediatric GISTs. It is commonly administered as an adjuvant treatment following R1 surgery in high-risk patients, and as neoadjuvant therapy to shrink tumors prior to surgical resection. Sensitivity to imatinib could also be tested to identify response rates before and during treatment. The natural killer (NK) cell receptor NKp30 isoforms and their soluble ligands predict the response to imatinib mesylate in metastatic GIST patients. Imbalanced NKp30 isoforms, particularly a low NKp30B/NKp30C ratio and low NKp30A expression, are linked to poor prognosis and an immunosuppressive tumor microenvironment [84]. Additionally, imatinib is beneficial in managing metastatic disease, helping to prolong overall survival. However, the rarity of pediatric GISTs complicates the establishment of robust clinical trials, and most of the data are based on adult clinical trials and guidelines [85,86,87].

For tumors resistant to imatinib, sunitinib serves as a second-line treatment. Sunitinib, a potent multikinase inhibitor used as a first-line therapy for metastatic renal cell carcinoma (mRCC) and “off-label” in pediatric oncology [88], is particularly effective in KIT wild-type GISTs. The adult equivalent of 50 mg/day should be an approx. 25 mg/m^2^ in children, but although initial data were promising [50,89], newer evidence suggests no evident tumor response apart from disease stabilization and a median disease-free progression of 5.8 months [90].

Regorafenib is another multikinase inhibitor used for imatinib-resistant tumors, usually as a third-line therapy. It targets multiple kinases, including the vascular endothelial growth factor receptor (VEGFR), TIE-2, Fibroblast growth factor receptor (FGFR), Platelet-derived growth factor receptors (PDGF-R), KIT, and RET. Long-term observations of patients with metastatic GISTs treated with regorafenib indicate notable benefits for those with primary KIT exon 11 mutations and SDH-deficient GIST [91]. While regorafenib is extensively used in adults, pediatric application is limited. Administered at 160 mg/day for three weeks each month, regorafenib has shown benefits, particularly in tumors with PDGFR mutations [6,92]. Preclinical models with mutated KIT oncogenes have demonstrated partial tumor regression, highlighting regorafenib’s potential for pediatric GISTs [93]. Several other multikinase inhibitors have been developed such as sorafenib [94], ponatinib [95,96], avapritinib, dasatinib [97], larotrectinib [98], or vandetanib. Most of them showed some results in adult mutated GISTs but failed to prove effective in pediatric/WT forms. Avapritinib, a potent KIT and PDGFRA (exon 18 mutation), used as a fourth-line therapy in adult-resistant GIST, failed to show a significant difference in outcomes compared to regorafenib, but the VOYAGER trial proved an interesting concept of monitoring the disease’s evolution during treatment using circulating tumor DNA (ctDNA) sample analysis [99,100]. Vandetanib is an oral multikinase inhibitor of VEGFR2, EGFR, and RET. In preclinical models, SDH deficiency results in elevated levels of hypoxia-inducible factor-1α (HIF1α). It was hypothesized that inhibiting the HIF1α-induced VEGF pathway would reduce the growth of dSDH GIST, but the clinical response failed again to be significant, with no tumor response or improvement in quality of life, just a prolonged state of stable disease in two out of nine patients as part of a phase II trial [83].

Another drug used to tackle the dSDH GIST challenge is the DNA methyltransferase inhibitor guadecitabine. Succinate dehydrogenase-deficient tumors display global hypermethylation, indicating that demethylating agents might be effective in their management. While there was a decrease in global methylation in peripheral blood mononuclear cells, no significant changes in metabolite concentrations or complete or partial tumor responses were observed, with some prolongation of the disease course in four out of nine patients [101].

WT GISTs were shown to increase insulin-like growth factor 1 receptor (IGF-1R) expression compared to KIT/PDGFRA-mutated GISTs. Preclinical data suggest that WT GIST cells may rely on IGF-1R, prompting the hypothesis that targeting IGF-1R could inhibit tumor growth. Linsitinib, an oral IGF-1R TKI, was therefore tested in patients with WT GIST [102]. Tumors were evaluated for IGF-1R expression, a loss of SDHA/B expression, and the activation of protein kinase B AKT and mammalian target of rapamycin (mTOR) as potential biomarkers of response. Lisitinib was well tolerated by patients, and although no objective response was seen, progression-free survival was improved by 9 months, offering increased potential for future therapies [103]. 

The medical treatment of GISTs differs significantly between adults and pediatric patients. Adult GISTs are primarily treated with targeted therapies like TKIs, such as imatinib, which are highly effective against tumors with KIT or PDGFRA mutations, with other TKIs like sunitinib and regorafenib used for resistant cases. In contrast, pediatric GISTs often show resistance to TKIs and conventional chemotherapies, and there is limited efficacy for standard immunotherapy. Ongoing research aims to identify effective targeted therapies for pediatric GISTs by focusing on alternative molecular pathways. Overall, while TKIs are the cornerstone of medical treatment for adult GISTs, pediatric GISTs necessitate a more tailored approach due to their distinct resistance patterns and genetic characteristics.

### 6.2. Surgical Treatment

Complete surgical resection is standard practice for both adult and pediatric/WT GIST. Because, even after total gastrectomy, recurrence is still at a certain risk, it is recommended to opt for limited resections (for example, wedge resection in the case of gastric GISTs), especially for lesions between 2 and 5 cm [104]. Challenging locations such as periampullary tumors or lesions at the gastroesophageal junction that present as larger than 5 cm may still be subject to complete gross-resection procedures that would necessitate reconstructive approaches such as Whipple or Billroth I. While, in adult subtypes, lymphatic or liver metastasis are rare, and thus lymphatic sampling is not recommended, in pediatric/WT GISTs, it might be necessary to opt for lymphatic dissection [19,105]. In adults, lymphadenectomy generally does not impact the disease relapse risk and may even be associated with increased mortality due to surgical complications, as adult GISTs infrequently spread to lymph nodes [106,107]. Conversely, pediatric GISTs are more aggressive and often involve lymphatic spreading, making lymphadenectomy a more common and potentially beneficial approach in this group [108] and linking lymph-node metastases to poorer survival outcomes and factors such as tumor size and mitotic activity [108,109].

Resection seems to still be advantageous for locally progressed or metastatic neoplasms, even in cases when R0 microscopic margins are not achieved [110], but overall survival (seen mainly in adult cohorts) is decreased [111]. There are a few different approaches to managing a positive microscopic margin following macroscopic full resection: watchful waiting, re-excision, and postoperative imatinib treatment, none of which were confirmed to be valid [21]. For patients who have a high risk of recurrence, adjuvant therapy with imatinib, 400 mg/day, for three years is the recommended course of action [85], although most WT/pediatric GIST will not present with a tumor response. Neoadjuvant treatment with imatinib for 6–12 months is advised for locally extensive tumors, with cytoreduction proven in certain studies, along with the same holdback of not having any benefit on WT/pediatric GISTs [112].

Tumor spill or rupture during surgery is a real concern, with some studies on adult patients revealing an up to 100% recurrence rate in ruptured tumors [112,113]; thus, several techniques have been used to prevent tumor dissemination during its handling. Although en-bloc open resection was considered the standard of care in the past, with some cohorts keeping the classic Billroth I technique as a go-to indication [114], minimally invasive procedures took the lead and presented as safe and feasible. 

#### Minimally Invasive Techniques

Laparoscopic wedge resection has evolved since 2008, when a group of surgeons and endoscopists developed the laparoendoscopic cooperative surgery (LECS) technique [115]. This novel approach offers the advantage of endoscopic transillumination and margin delimitation for a more precise tumor-free margin and favorable postoperative anatomy, using electrocautery endoscopic and endoscopic dye-margin delimitation, which can be easily followed from the laparoscopic part with wedge resection. This procedure presents with minimal intraoperative bleeding (10–31 mL) [116,117], as well as rapid oral intake (within 24 h post-procedure) and discharge (48 h postop.), with a surgical procedure timeframe evolving from 253 min at the development to 98.5 min on average, as described by Pulido et al. [118]. Furthermore, because of facing the possible complication of abdominal-cavity dissemination via laparoscopic tumor extraction, other hybrid subtypes were developed in which the resection piece is pulled out endoscopically [119]. Inverted LECS involves marking the tumor endoscopically, making an incision laparoscopically, and then inverting the tumor into the gastric lumen for safe resection. This approach helps ensure clear margins and minimizes the contamination of the peritoneal cavity with gastric contents. Closed LECS is a variation of the standard LECS technique performing an endoscopic dissection around the tumor within the submucosal layer, using laparoscopy to mark the resection line on the serosal surface in accordance with submucosal dissection, suturing the seromuscular layer while inverting the marked lesion into the stomach, conducting a circumferential seromuscular dissection endoscopically, and retrieving the tumor orally, reducing the risk of infection and maintaining the integrity of the gastric wall [117]. Classical LECS, CLEAN-NET, and NEWS share a common concept but differ in their specific procedures. These differences include whether they use exposure or non-exposure techniques, whether the tumor is inverted into the lumen or outside, whether the tumor is retrieved orally or through the abdominal cavity, and whether the endoscopist or the laparoscopic surgeon plays the dominant role [120]. However, there are still limitations that hinder the widespread use of these techniques, including challenges with tumor localization, tumor size, and local invasion. Additionally, it is important to note that, as of the time of this review, there have been no reported procedures conducted on pediatric patients [121,122].

## 7. Follow-Up and Survival

The recommended follow-up for pediatric gastrointestinal stromal tumors (GISTs) involves regular clinical evaluations, imaging studies, and genetic testing. Initial assessments typically include comprehensive imaging such as MRI or computer tomography scans to monitor tumor size and detect any metastases. Long-term follow-up should include periodic imaging every 3–6 months for the first few years, and then annually if a stable condition is achieved, to monitor for recurrence or progression. Additionally, patients should receive ongoing assessments of treatment efficacy and potential side effects, along with supportive care and counseling for themselves and their families [5,9]. 

One of the most intriguing aspects of comparing pediatric to adult GISTs is the difference in survival rates. Despite pediatric GISTs exhibiting characteristics of an aggressive malignancy—such as high mitotic index, invasive behavior, a tendency to metastasize, and increased resistance to standard chemotherapy and immunotherapy—pediatric GIST patients actually show better survival outcomes than adults. Specifically, the 5-year GIST-specific survival (GSS) rate for pediatric patients is 83.3%, compared to 75.4% for adults, while the 5-year overall survival (OS) rate is 82.4% for pediatric patients, versus 67.1% for adults. This paradox highlights that, despite the more aggressive appearance of pediatric GISTs, they can sometimes exhibit a less aggressive clinical course compared to their adult counterparts [123].

## 8. Case Report

**Figure 2:**
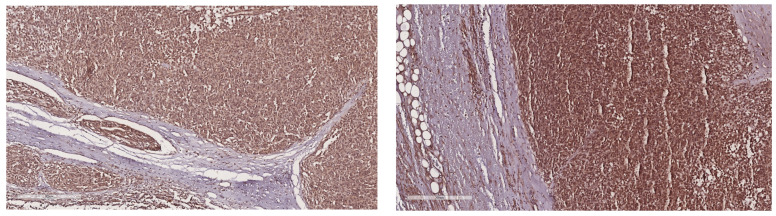
SDH immunohistochemistry—normal distribution of SDH in the bioptic tumor tissue.

**Figure 3:**
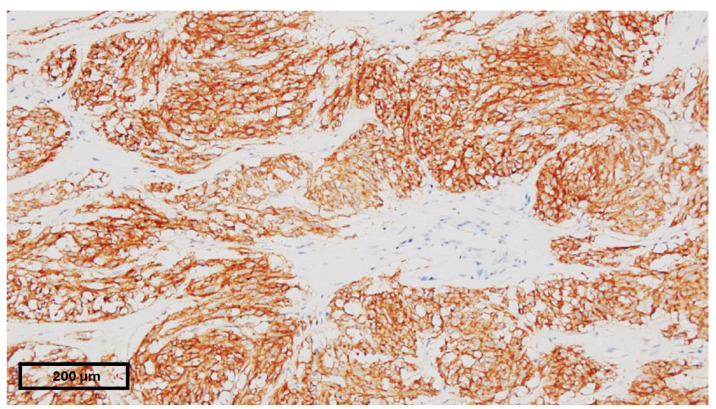
CD117-positive biopsy.

**Figure 4:**
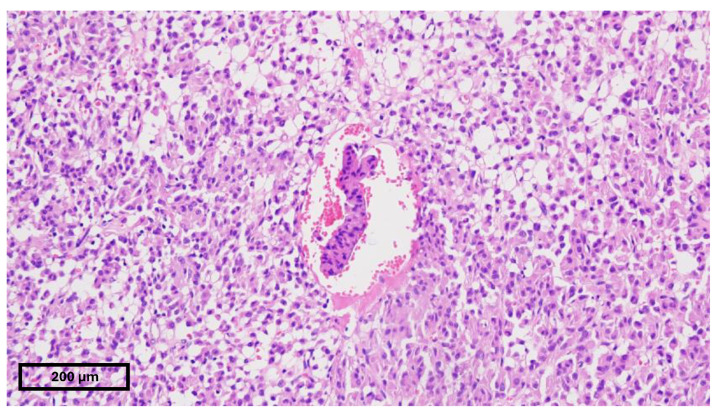
Vascular invasion—hematoxiline–eozine 20×.

A 15-year-old female presented to our emergency department with severe abdominal pain and hemodynamic instability. The lab findings revealed a concomitant normocytic, hypochromic anemia characterized by a hemoglobin level of 7.4 g/dL (normal range: 11.8–15.7 g/dL), a red blood cell count of 3.19 × 10^6^/mm^3^ (normal range: 4.2–5.4 × 10^6^/mm^3^), and hematocrit of 25.8% (normal range: 34–45%). The mean corpuscular volume (MCV) was 80.9 fL (normal range: 73–90 fL), with a decreased mean corpuscular hemoglobin concentration (MCHC) of 28.7 g/dL (normal range: 32–36 g/dL) and mean corpuscular hemoglobin (MCH) of 23.2 pg (normal range: 25–31 pg). Additionally, there was an increased red blood cell distribution width coefficient of variation (RDW-CV) at 19.3% (normal range: 11.5–16%). She had been diagnosed with a gastrointestinal stromal tumor (GIST) confirmed with a biopsy at a pediatric surgery clinic and was undergoing treatment with imatinib. Due to suspected intraabdominal bleeding from the tumor, she was referred to our hospital for potential interventional radiology treatment. However, the embolization attempt was unsuccessful, necessitating urgent surgical intervention by our general surgery team in collaboration with the pediatric surgery team. Preoperative computed tomography revealed hemoperitoneum, a gastric mass consistent with a GIST, and multiple hepatic metastases. During laparotomy, a significant hemoperitoneum of 2.5 L was detected, with the tumor-infiltrating the mesocolon and gastrohepatic ligament. The bleeding originated from within the ulcerated tumor. Surgical management included ligation of the left gastric artery, partial gastrectomy with omega loop reconstruction, and left hemicolectomy. Postoperative tomography indicated a distended stomach and dilated ileal and jejunal loops, which were managed with nasogastric tube placement and steroids. The patient was subsequently transferred to the intensive care unit (ICU), where her recovery was favorable. She quickly resumed oral nutrition and was later transferred back to the pediatric oncology department for continued treatment. An anatomopathological examination revealed tumor invasion beyond the posterior gastric wall and into the mesocolon. The resected specimen contained multiple tumoral nodules, predominantly composed of epithelioid and fusiform cells. Immunohistochemical staining was positive for CD117, with increased mitotic activity, all indicative of a GIST phenotype. Subsequent staining for succinate dehydrogenase subunit B (SDHB) was positive, ruling out SDH deficiency (Figure 2, Figure 3 and Figure 4). Currently, she is undergoing cycles of sunitinib. Although there has been no objective tumor response, imaging shows progression-free survival.

### Case Discussion

**Figure 5:**
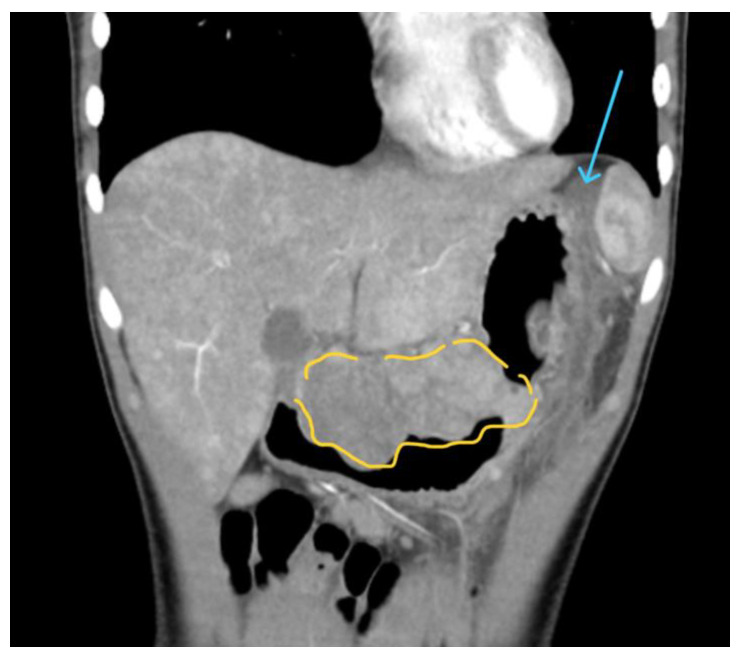
Gastric tumor-GIST seen in CT scan before biopsy was obtained. In yellow you can see a large tumor mass. The blue arrow points to ascites present in the abdomen.

**Figure 6:**
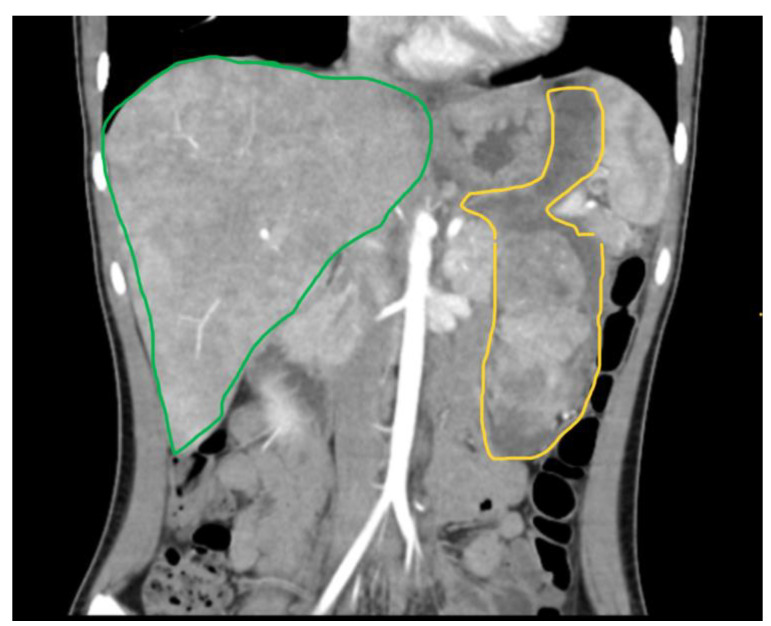
A large peritoneal tumor mass (outlined in yellow) located in the left upper hemiabdomen, with a solid component that absorbs iodine located distally and a liquid component. The liver (outlined in green) shows the diffuse pathological uptake of the contrast substance at the periphery.

**Figure 7:**
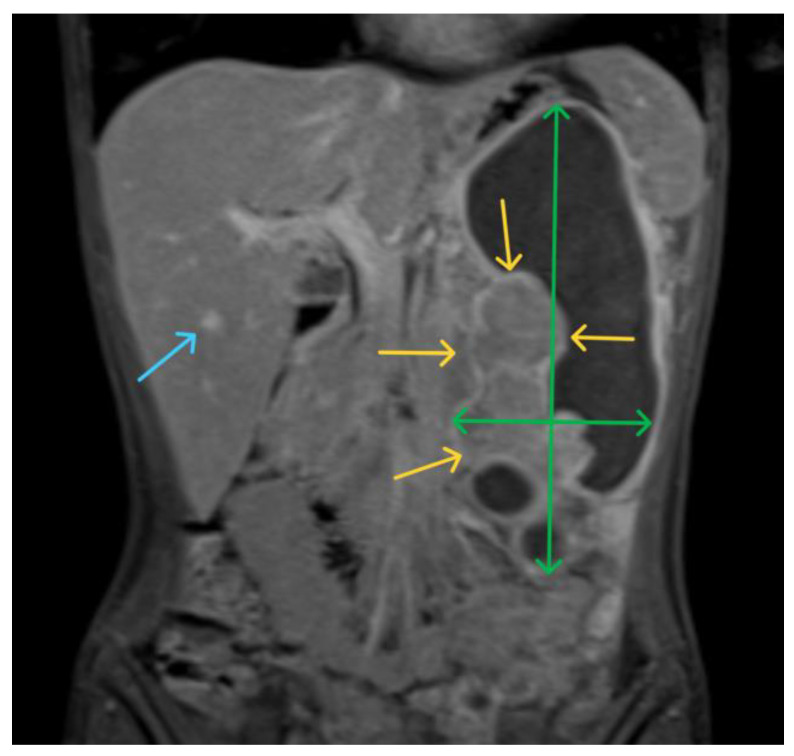
MRI T1 sequence of the abdomen, coronal section in the venous phase: Secondary hepatic lesion with gadolinium uptake, subcentimetric (blue arrow). Tumor mass (green arrows) with a solid component showing gadolinium uptake (yellow arrows) and a liquid component.

The initial diagnosis in the pediatric population, particularly at smaller, non-specialized centers, poses significant challenges. This patient exhibited symptoms of vomiting, fatigue, and anemia for nine months before her referral to a specialized pediatric surgery center. During this period, she was diagnosed with macrocytic anemia of unknown etiology, which was unresponsive to treatment. Upon admission to the pediatric surgery clinic, imaging studies, including ultrasound, computer tomography, and MRI (Figure 5, Figure 6 and Figure 7), revealed a gastric tumoral mass with both solid and liquid components, alongside secondary hepatic lesions suggestive of metastases. The aggressive nature of the tumor was evident from these imaging findings, yet a definitive diagnosis could not be established without a biopsy, necessitating a surgical intervention for tissue sampling.

Given the tumor’s extensive invasion of the anterior gastric wall, the lesser curvature of the stomach, the gastrohepatic ligament, and the presence of hepatic metastases, coupled with its propensity for bleeding, no curative surgery was pursued initially. This approach, although based on clinical intuition in the absence of a confirmed GIST diagnosis at the time, aligns with international guidelines advising against radical surgery for invasive GISTs or those with a high risk of incomplete resection (R1) [8,110]. Debulking or cytoreductive surgical procedures are generally not recommended for GISTs, as R2 resections (with macroscopic residual disease) do not provide a survival advantage. These procedures are typically reserved for palliative or emergency situations, such as bowel obstruction or hemorrhage, where debulking may be necessary to alleviate symptoms. In such cases, as demonstrated here, the primary goal is to address immediate complications, rather than improve long-term survival [124].

Major complications of GISTs, though relatively uncommon, can be severe and include bowel obstruction, intussusception, hemorrhage, and perforation, often necessitating surgical intervention. GISTs typically present acutely with upper gastrointestinal bleeding (melena or hematemesis) in 48.9% of cases, intestinal obstruction in 28.3%, or peritonitis in 7.6% [125,126]. In our case, we initially attempted embolization to manage the bleeding due to its less-invasive nature. Unfortunately, this approach failed, and surgical intervention was required.

A histopathological analysis of the biopsy confirmed the diagnosis of GIST, characterized by fusiform cells with focal epithelioid features, eosinophilic cytoplasm, areas of perinuclear vacuolization, moderate nuclear atypia, and a mitotic count of less than 5 per 50 high power fields (HPFs), with no evidence of necrosis. These lesions were situated within the smooth muscle. Notably, a subsequent biopsy following emergency surgery (indicated earlier in Figure 1) indicated an increase in the mitotic index, suggesting a possible transformation towards greater aggressiveness. This transformation could also be explained by the influence of the tumor microenvironment after being exposed to certain chemotherapeutics with questionable effectiveness, as seen in other types of cancer [127].

An immunohistochemical analysis showed patchy SDHB expression with preserved expression of mismatch repair (MMR) proteins (MLH1, MSH2, MSH6, and PMS2). Genetic testing utilizing the Illumina TruSight™ Oncology 500 (TSO500) next-generation sequencing assay reported no variants of strong clinical significance (Tier I). However, amplifications of the FGF3 gene with three copies were identified as variants of potential clinical significance (Tier II). This is of particular interest because several mutations of the FGF and FGFR family were associated with quadruple-negative WT GISTs, as seemed to be the case here [26,128,129]. The FGF/FGFR signaling pathway has emerged as significant in the oncogenesis of various GIST subsets, including those with SDH deficiencies or that are quadruple-WT, exhibiting deregulated FGF/FGFR signaling [81]. This pathway is implicated in tumor growth, survival, and resistance to therapies such as imatinib. Multi-target tyrosine kinase inhibitors (TKIs) like regorafenib, which also target FGFR, are being used and evaluated for their effectiveness in GISTs, particularly in cases resistant to standard treatments. Inhibitors specifically targeting FGFR, such as dovitinib, have shown promise in clinical trials, providing alternative therapeutic strategies for GIST patients [130,131]. The efficacy of these treatments in different molecular subsets, including those without KIT or PDGFRA mutations, is an area of ongoing research. The interaction between KIT and FGFR pathways plays a critical role in the development of resistance to conventional treatments in GISTs. Studies have shown that the inhibition of FGFR can restore sensitivity to imatinib in resistant GIST cells, highlighting the potential for combination therapies [132].

Pediatric gastrointestinal stromal tumors present unique challenges in diagnosis, treatment, and follow-up due to their rarity and distinct molecular profiles compared to adult GISTs. The case of the 15-year-old female patient underscores the complexity of managing pediatric GISTs, particularly when they present emergently with severe symptoms such as intra-abdominal bleeding. This case highlights the importance of a multidisciplinary approach involving timely surgical intervention and the use of targeted therapies like tyrosine kinase inhibitors. Regular follow-up with advanced imaging and genetic testing is crucial to monitor disease progression and the treatment response. Emergency presentations, although less common, emphasize the need for readiness to address acute complications in pediatric GIST patients, ensuring prompt and effective treatment to improve outcomes.

## 9. Conclusions

Pediatric gastrointestinal stromal tumors (GISTs) exhibit distinct molecular and clinical profiles compared to adult GISTs, necessitating tailored diagnostic and therapeutic strategies. Unlike adult GISTs, which commonly harbor KIT or PDGFRA mutations, pediatric cases often lack these mutations, and instead, they may display abnormalities in the SDH complex or other unique genetic markers. These differences complicate the use of standard treatments like imatinib, which are highly effective in adults. Pediatric GISTs also tend to exhibit an indolent but unpredictable clinical course, making regular monitoring and comprehensive management crucial. Multidisciplinary approaches, incorporating pediatric oncologists, surgeons, pathologists, and genetic counselors, are essential for optimizing outcomes. Future research should focus on elucidating the distinct molecular pathways involved in pediatric GISTs to develop more effective targeted therapies. The rarity and complexity of these tumors underscore the need for specialized care and ongoing investigation to improve the prognosis and quality of life for affected children.

## Data Availability

Data sharing is not applicable (only appropriate if no new data are generated or the article describes entirely theoretical research).
